# NDM-1–producing *Klebsiella pneumoniae*, Croatia

**DOI:** 10.3201/eid1803.1103890

**Published:** 2012-03

**Authors:** Annarita Mazzariol, Zrinka Bošnjak, Piero Ballarini, Ana Budimir, Branka Bedenić, Smilja Kalenić, Giuseppe Cornaglia

**Affiliations:** University of Verona, Verona, Italy (A. Mazzariol, P. Ballarini, G. Cornaglia);; University of Zagreb, Zagreb, Croatia (Z. Bošnjak, A. Budimir, B. Bedenić, S. Kalenić)

**Keywords:** NDM metallo-beta-lactamase, carbapenem resistance, Klebsiella pneumoniae, bacteria, Croatia

**To the Editor:** The novel metallo-β-lactamase named New Delhi metallo-β-lactamase (NDM-1) was identified from *Klebsiella pneumoniae* and *Escherichia coli* isolates in Sweden from a patient previously hospitalized in India (*1*). NDM-1 is spreading rapidly worldwide to nonclonally related isolates, many of which are directly or indirectly tracked to the Indian subcontinent (*2*). A carbapenem-resistant *K. pneumoniae* strain, KLZA, was isolated in May 2009 from the culture of a blood sample from of a 40-year-old man on the day after his admission to a surgical intensive care unit of the Clinical Hospital Center in Zagreb, Croatia. The patient had been transferred after 5 days of hospitalization in Bosnia and Herzegovina following a car accident. The clinical history mentioned antimicrobial drug treatment that did not include carbapenems (gentamicin, metronidazole, and ceftriaxone) and no link to the Indian subcontinent. Antimicrobial drug susceptibility testing was performed by Vitek2 (bioMérieux, Marcy-l’Etoile, France) and broth microdilution and interpreted according to the latest documents from the European Committee on Antimicrobial Susceptibility Testing (www.eucast.org/clinical_breakpoints/, version 1.1).

The strain proved resistant to imipenem and meropenem, to all broad-spectrum cephalosporins, and to aminoglycosides and susceptible to ciprofloxacin and tigecycline (Table). We checked for *bla*_VIM_, *bla*_IMP_, *bla*_SPM_, *bla*_GIM_, *bla*_SIM_, and *bla*_NDM_ resistance genes by using PCR. A PCR product was obtained only with the NDM primers, after being purified (QIAquick PCR Purification Kit, QIAGEN, Hilden, Germany), its sequence showed 100% identity with *bla*_NDM-1_.

**Table:** MIC of the KLZA strain of *Klebsiella pneumoniae* and its transconjugant and recipient

Antimicrobial drug	MIC, mg/L		
	*K. pneumoniae* KLZA	*Escherichia coli* J53	*E. coli* T1
Imipenem	8	<0.06	4
Meropenem	8	<0.06	4
Ertapenem	16	<0.06	8
Ceftazidime	>128	<0.06	128
Cefotaxime	>128	<0.06	32
Cefepime	32	<0.06	64
Aztreonam	>128	0.25	>128
Ciprofloxacin	0.5	<0.06	0.12
Gentamicin	8	0.25	0.25
Amikacin	16	0.5	0.5
Tigecycline	1	0.25	0.25
Colistin	<0.5	<0.5	<0.5

Strain genotyping was performed by multilocus sequence typing to determine the sequence type (ST) of the isolate and to establish a comparison with previously reported NDM-1–producing isolates. Allelic numbers were obtained on the basis of sequences of 7 housekeeping genes at www.pasteur.fr/recherche/genopole/PF8/mlst/Kpneumoniae.html. Multilocus sequence typing identified *K. pneumoniae* KLZA as an ST25 strain, which significantly differs from the ST14 type found in the index NDM-1–producing strain and from other isolates originating from India (*1*) and then in other countries. ST25 *K. pneumoniae* was also found in *K. pneumoniae* isolates in Geneva (*3*). Other *K. pneumoniae* STs harboring NDM-1 were ST15, ST16, and ST147 (*4*–*7*).

Resistance was transferred by conjugation to *E. coli* J53, with selection based on growth on agar in the presence of ceftazidime (10 mg/L) and azide (100 mg/L). The conjugant T1 showed resistance to β-lactams, including all carbapenems, as well as decreased susceptibility to ciprofloxacin.

The KLZA strain and its transconjugant harbored other determinant of resistance, namely *bla_CTX-M-15_*, *bla_CMY-16_*, and *qnrA6*. Plasmid incompatibility groups, determined by a PCR-based replicon typing method, belonged to the incA/C replicon type.

This report of an NDM-1–producing *K. pneumoniae* in Croatia adds to those of other cases in patients from patients hospitalized in the Balkan area. The patient in this report had no apparent link to the Indian subcontinent.

In a survey conducted by the European Centre for Disease Prevention and Control to gather information about the spread of NDM-1–producing *Enterobacteriaceae* in Europe and reporting cases from 13 countries during 2008–2010, five of the 55 persons with known travel histories had traveled to the Balkan region during the month before diagnosis of their infection: 2 to Kosovo and 1 each to Serbia, Montenegro, and Bosnia and Herzegovina. All had received hospital care in Balkan countries because of an illness or accident that occurred during the journey (*7*). Two of the latter cases (*4*,*8*) and a case from Germany (*9*) were subsequently published. No patient had any apparent link to the Indian subcontinent.

Although the way NDM-1 isolates might have been imported to western Europe not only from the Indian subcontinent but also from Balkan countries (*10*) has been highlighted, awareness of western Europe as a possible area of endemicity remains limited. The aforementioned report from Germany, although recognizing that the patient had been repatriated after hospitalization in Serbia, declared “no evidence about contact with people from regions where NDM-enterobacteria are endemic” (*9*). This limited awareness shows the threat of neglecting to screen patients who are transferred from countries thought not to be at risk for NDM-1. Furthermore, it means that specimen are not sent to the local reference laboratories and recognized as positive for NDM-1, thus permitting wide dissemination of NDM-1–producing enterobacteria in the community (*4*). The accumulating evidence of NDM-1 from the Balkan area could suggest a possible multifocal spread of this enzyme, with the Balkans as a possible second area of endemicity, in addition to the Indian subcontinent, and prompts for widespread epidemiologic surveillance.
